# Characterization of *Leishmania donovani* MCM4: Expression Patterns and Interaction with PCNA

**DOI:** 10.1371/journal.pone.0023107

**Published:** 2011-07-29

**Authors:** Neha Minocha, Devanand Kumar, Kalpana Rajanala, Swati Saha

**Affiliations:** 1 Department of Microbiology, University of Delhi South Campus, New Delhi, India; 2 National Institute of Immunology, New Delhi, India; University of Minnesota, United States of America

## Abstract

Events leading to origin firing and fork elongation in eukaryotes involve several proteins which are mostly conserved across the various eukaryotic species. Nuclear DNA replication in trypanosomatids has thus far remained a largely uninvestigated area. While several eukaryotic replication protein orthologs have been annotated, many are missing, suggesting that novel replication mechanisms may apply in this group of organisms. Here, we characterize the expression of *Leishmania donovani* MCM4, and find that while it broadly resembles other eukaryotes, noteworthy differences exist. MCM4 is constitutively nuclear, signifying that, unlike what is seen in *S.cerevisiae*, varying subcellular localization of MCM4 is not a mode of replication regulation in *Leishmania*. Overexpression of MCM4 in *Leishmania* promastigotes causes progress through S phase faster than usual, implicating a role for MCM4 in the modulation of cell cycle progression. We find for the first time in eukaryotes, an interaction between any of the proteins of the MCM2-7 (MCM4) and PCNA. MCM4 colocalizes with PCNA in S phase cells, in keeping with the MCM2-7 complex being involved not only in replication initiation, but fork elongation as well. Analysis of a LdMCM4 mutant indicates that MCM4 interacts with PCNA via the PIP box motif of MCM4 - perhaps as an integral component of the MCM2-7 complex, although we have no direct evidence that MCM4 harboring a PIP box mutation can still functionally associate with the other members of the MCM2-7 complex- and the PIP box motif is important for cell survival and viability. In *Leishmania,* MCM4 may possibly help in recruiting PCNA to chromatin, a role assigned to MCM10 in other eukaryotes.

## Introduction

Eukaryotic DNA replication involves the licensing and activation of multiple origins. Origins are licensed by the assembly of pre-replication complexes (pre-RCs) in G1 phase [1]–[3], a process involving the ordered loading of ORCs 1-6, Cdc6, Cdt1, MCM2-7, and MCM10. At the G1/S transition, Cdc7/Dbf4 and Cdk2/cyclin E kinase activity transform pre-RCs into pre-initiation complexes. GINS, Sld2, Dpb11 and Cdc45 associate with the complexes to trigger origin activation, and with the recruitment of the elongation machinery, DNA synthesis commences [4], [5]. While replication has been extensively examined in higher eukaryotes and yeasts, the pre-replication and replication apparatus of protozoans remains largely uninvestigated, with most reports being from studies in *Plasmodium falciparum* [6]-[9] and *Tetrahymena thermophila* [10]–[12].

The trypanosomatid *Leishmania* causes the group of diseases collectively called Leishmaniasis. Leishmaniasis occurs in three main forms – cutaneous, subcutaneous and visceral, and different *Leishmania* species cause different forms of the disease. Leishmaniasis is prevalent in 88 countries across the globe, and inflicts mostly people of the economically weaker sections of society. Every year ∼1.6 million new cases are reported, of which about 500,000 are cases of visceral leishmaniasis (VL). Around 90% of the cases of VL occur in South Asia and East Africa. VL can be fatal if not treated early and appropriately, and several research groups are engaged in investigating the biology of the causative pathogens of VL, with the aim of developing more effective means of therapeutic intervention. *Leishmania donovani* is one of the causative agents of VL, prevalent in Sudan and the Indian subcontinent.

*Leishmania* species cycle between two hosts – the insect sandfly, and the mammalian host. In the insect host they exist as flagellate promastigotes. The promastigotes remain attached to the wall of the anterior region of the midgut, as non-infective procyclic forms in the early stages. As the parasites further develop, they detach from the midgut and migrate to the salivary glands. These metacyclic forms are infective. When the insect bites the mammalian host the promastigotes are released into the mammalian host's bloodstream where they are taken up by host macrophages. Within the macrophages they transform into aflagellate amastigotes, and propagate. The amastigotes are transferred to the insect host with a bloodmeal where they transform into promastigotes again. Microarray analysis reveals the absence of stage-specific putative DNA replication proteins in *Leishmania* promastigotes and amastigotes [13], not unexpectedly, as both forms of the parasite reproduce asexually by binary fission. The components of pre-RCs are conserved from yeast to mammals, with the basic mechanisms of DNA replication being similar. However, based on their annotated genome sequences [14]–[17], while the replication machinery of trypanosomatid nuclear DNA appears to largely resemble that of higher eukaryotes, several key players are absent. Only one ORC ortholog – ORC1- is present; no orthologs of Cdt1, Dbf4 or Cdc7 are apparent. Orthologs of MCM2-7 and Cdc45 have been annotated. Investigations in the area of trypanosomatid nuclear DNA replication have thus far largely centered around the ORC1 protein. The ORC1 in *Leishmania major* is nuclear throughout the cell cycle [18], and knockdown of ORC1 in *T.brucei* results in anucleate cells [19]. The presence of replication foci has been demonstrated in *Leishmania donovani,* and PCNA serves as a marker for these factories [20].

MCM2-7, originally identified as *Saccharomyces cerevisiae* mutants defective in **m**ini**c**hromosome **m**aintenance [21], are grouped together based on sequence similarity, being defined by a 200 amino acid MCM box domain. MCM2-7 loading at origins is promoted by ORC-Cdc6 mediated ATP hydrolysis in *S. cerevisiae* [22]. As replication transitions from initiation to elongation phase the MCM2-7 are believed to play the role of replicative helicase. The core complex of MCM4/6/7 from *S. cerevisiae, S. pombe*, mouse and human cells, exhibits helicase activity *in vitro* [23], [24], and the heterohexamer MCM2-7 from *S. cerevisiae* has been demonstrated to display helicase activity *in vitro* as well [25]. The role of other accessory proteins in mediating the complex's helicase activity *in vivo* has been implicated in *Xenopus*, with Cdc45 one of the favourite candidates [26].

This study is the first report characterizing MCM4 in any of the trypanosomatids. The *Leishmania donovani* gene has been cloned and the expression patterns of the protein examined at different stages of the *Leishmania* parasite as well as at different phases of the cell cycle. Unlike in *Saccharomyces cerevisiae*, LdMCM4 remains nuclear throughout the cell cycle, ruling out nuclear export in mid to late S phase as a mode of replication regulation. Overexpression of MCM4 in *Leishmania* promastigotes results in cells reaching G2/M phase faster than usual, implicating a possible role in DNA replication and cell cycle progression. The protein interacts with PCNA *in vitro*, and co-localizes with PCNA in S phase cells. The interaction occurs via the PIP box motif of MCM4, and the importance of the MCM4 PIP box motif is underlined by the fact that cells overexpressing mutated MCM4 protein that cannot interact with PCNA, display decreased viability. These data signify that while the mechanism of DNA replication in *Leishmania* is generally conserved with that of higher eukaryotes, there are significant novel features in the process as well, pointing to the importance of studying replication not only in model eukaryote systems, but in non-conventional organisms as well.

## Materials and Methods

### Leishmania cultures and cell synchronization

*Leishmania donovani* 1S promastigotes were cultured as described [18]. *L. donovani* cultures were synchronized by treating exponentially growing cultures (2–3×10^7^ cells/ml) with 5 mM hydroxyurea for 8 h, and released into drug-free M199 medium, as described [27].

### Flow cytometry analysis

1–2×10^7^ promastigotes were harvested, washed with PBS, fixed in 30% PBS/70% methanol overnight at 4°C, collected by centrifugation, washed with PBS, and stained in PBS containing propidium iodide (100 µg/ml) and RNase (100 µg/ml) at 37°C for 30 min. Flow cytometry analysis was carried out using a BD FACSCalibur flow cytometer and the CellQuest Pro software (BD Biosciences). G1, S and G2/M phases are indicated on the histograms by the gates M1, M2 and M3 respectively, which were derived from the data using the CellQuest Pro software.

### Genomic DNA isolation and cloning

Genomic DNA was isolated as described [20]. The MCM4 gene product amplified from *L.donovani* genomic DNA using *Phu* DNA polymerase (Finnzymes) and primers MCM4-F and MCM4-R (Table S1), was cloned into pENTR/D-TOPO (Invitrogen) for sequencing, and subcloned for expression in *E.coli* as detailed in Supporting Information S1. The amplicon was cloned into pXG-/GFP+ (a kind gift from Prof. Beverley) and pXG-/GFP+/FLAG (described in Supporting Information S1), creating plasmids pXG/MCM4 - GFP and pXG/MCM4-FLAG, for expression in *Leishmania*.

### Site-directed mutagenesis

MCM4/PIP box mutant was made by overlap PCR. MCM4-GFP-F and MCM4-PIP-R primers amplified the N-terminal part of the mutant gene, and MCM4-PIP-F and MCM4-GFP-R (Table S1) amplified the C-terminal part. The products of these two PCRs were used as template for amplification of the full length MCM4/PIP mutant gene using MCM4-GFP-F and MCM4-GFP-R primers. The amplicon was cloned into pXG-/GFP+ and pXG-/GFP+/FLAG to create plasmids pXG/MCM4/PIP-GFP and pXG/MCM4/PIP-FLAG for expression in *Leishmania*.

### Recombinant protein expression and purification

MCM4 expression was induced from pASK-MCM4 in BL21 Codon Plus (Stratagene) cells, at 16°C for 16–18 hours. The expressed LdMCM4 was insoluble. MCM4 was solubilized using 8 M urea in 100 mM Tris.Cl (pH 8.0), 150 mM NaCl, 1 mM EDTA and dialyzed step-wise to 0 M urea. The protein was purified by Strep-Tactin II resin affinity chromatography (IBA BioTAGnology) according to the manufacturer's instructions.

### Preparation of Leishmania extracts

*Leishmania* whole cell extracts were prepared using the M-PER kit (Pierce Biotechnology) according to the manufacturer's instructions. *Leishmania* cytosolic and nuclear extracts were made as described in Supporting Information S1. *Leishmania* procyclics and metacyclics were separated as described in Supporting Information S1.

### Treatment of Leishmania with hydroxyurea, aphidicolin and UV radiation

Logarithmically growing *Leishmania* promastigote cultures at a cell density of 3×10^7^ cells/ml were incubated with 5 mM hydroxyurea for 8 h. Cells were then harvested and extracts made for Western blot analysis. Logarithmically growing promastigote cultures at a cell density of 2×10^7^ cells/ml were treated with 2 µM aphidicolin for 24 h prior to harvesting for Western blot analysis of whole cell lysates. For treatment with UV radiation, promastigotes at a cell density of 4×10^7^ cells/ml were irradiated in a six-well cluster dish, with a hand held UV lamp (254 nm) for 20 min. Cells were then harvested for analysis.

### Leishmania transfections

*Leishmania* promastigotes were transfected as described [28]. G418 (100 µg/ml) was added 42 h after transfection. MCM4-GFP expression was analyzed 6–8 days after drug-induced selection pressure. At this stage, more than 80–85% of the surviving cells were MCM4-GFP positive. Flow cytometry analyses were carried out two weeks after transfections or later, by which time more than 95% of the cells were MCM4-GFP positive. Pulldown experiments with MCM4-FLAG were performed two weeks (or later) after transfections. For making clonal lines, immediately after electroporation (1×10^8^ cells) the cells were transferred into 10 ml M199 for recovery, and after 42 h, the cell aggregates were removed by centrifugation and the rest of the cells plated on semi-solid M199 medium containing G418 (50 µg/ml). Isolated colonies were apparent after 10–12 days, and these were expanded step-wise in M199 medium containing G418 (100 µg/ml). Clonal lines were maintained in the presence of drug.

### Pull-down assays and immunoprecipitations

30–40 µg His-PCNA (overexpressed and purified from pASK-PCNA by Strep-Tactin chromatography) was incubated with Talon metal affinity resin beads (BD Biosciences) equilibrated with 50 mM Tris.Cl (pH 8.0) containing 300 mM NaCl, using a nutator mixer, at 4°C for 1 h. After removing unbound PCNA by two successive washes, whole cell extract from 1×10^9^ exponentially growing promastigotes [that had been treated with 20 U of DNase I (New England Biolabs, USA) for 15 min with mixing using a nutator mixer during isolation of extract], was added in the presence or absence of 500 µM ATPγS. The mix was incubated at 4°C for 2 h using a nutator mixer. In identical control binding reactions, equivalent amount of extract was added to resin beads not carrying bound His-PCNA. Unbound extract was removed by 5–6 successive washes with 50 mM Tris.Cl (pH 8.0) containing 1 M NaCl. The immobilized PCNA (along with any interacting proteins) was eluted with 200 mM imidazole by incubation using a nutator mixer for 5 min at 4°C. One-sixth of the eluate was analyzed by Western blotting.

For immunoprecipitation of FLAG-tagged proteins, DNase I-treated whole cell lysates (prepared as described above) made from 2×10^9^ cells that were transfected with MCM4-FLAG plasmid constructs (wild type or PIP mutant), were incubated with FLAG M2 agarose beads (Sigma Aldrich, USA) equilibrated with 1X PBS, at 4°C using a nutator mixer, for 2 h. The beads were washed extensively with 1X PBS containing 0.2% Triton X-100, to remove unbound and non-specifically bound proteins. The beads were then boiled in 3X SDS sample loading buffer, and analyzed by SDS-PAGE followed by Western blotting.

### Analysis for phosphorylation status

MCM4-FLAG (wild type) was immunoprecipitated from 2×10^8^ cells using FLAG M2 agarose beads, washed extensively, and the beads boiled in 3X SDS sample loading buffer, as described above. The sample was divided into three equal parts, and each part was resolved along with recombinant MCM4 on a 10% SDS-PAGE, followed by Western blotting. All three parts along with recombinant protein, were resolved on the same gel along with protein marker, so as to be able to align the different bands with respect to each other after Western blot analysis using the different antibodies. One part was analyzed for MCM4-FLAG expression with anti-FLAG antibody (Sigma Aldrich, 1∶5000 dilution), the second part for phoshorylated serine residues with anti-phosphoserine antibody (Sigma Aldrich; 1∶2000 dilution), and the third part for MCM4 with anti-MCM4 antibody (1∶1000 dilution).

The experiment was similarly performed for analysis of phosphorylated threonine residues (anti-phosphothreonine antibodies from Cell Signaling Technology; 1∶2000 dilution) and phosphorylated tyrosine residues (anti-phosphotyrosine antibodies from Cell Signaling Technology; 1∶1000 dilution).

### Immunofluorescence analysis

MCM4-GFP expression was studied by examining direct fluorescence of promastigotes harboring MCM4-GFP as described [18]. Expression of PCNA was examined as described [20]. For examining co-localization of MCM4-GFP with PCNA, exponentially growing promastigotes harboring MCM4-GFP were synchronized by an 8 h hydroxyurea block and analyzed 3 hours after release. Imaging of nuclei was done with an inverted motorized confocal microscope (LSM 510; Carl Zeiss MicroImaging, Inc.) (100X objective). For colocalization examinations, Z stack images of cells were collected.

## Results

### MCM4 is expressed in actively proliferating Leishmania promastigotes

**Figure 1:**
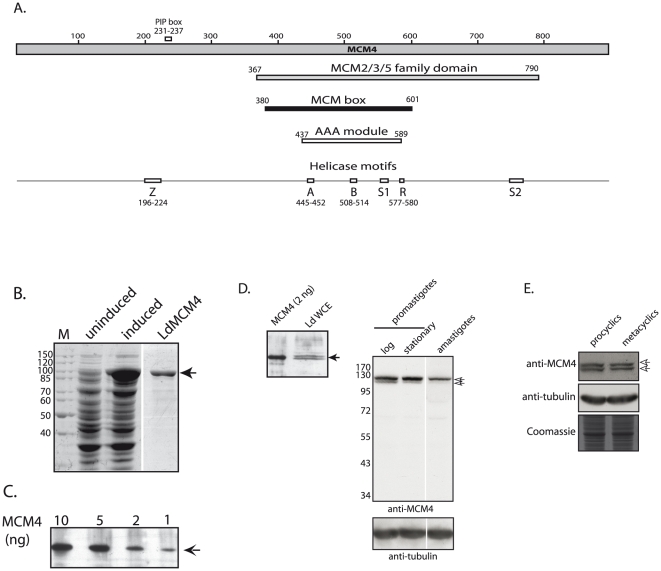
MCM4 expression in *Leishmania*. **A**. Schematic representation of the conserved domains in LdMCM4. PIP box - PCNA interacting protein motif; A – Walker A motif; B – Walker B motif; R – arginine finger; Z – zinc finger; S1 – sensor 1; S2 – sensor 2. **B**. SDS-PAGE (10% PAGE) analysis of overexpressed and purified recombinant LdMCM4 (2 ug; ∼97.2 kDa). **C**. Western blot analysis of recombinant LdMCM4 with mouse anti-MCM4 antibody (1∶5000 dilution). **D**. Western blot analysis (10% SDS-PAGE) using anti-MCM4 antibodies (dilution of 1∶1000). First panel - lane 1, recombinant MCM4; lane 2, *L. donovani* whole cell extracts. Second panel - Whole cell extracts from *Leishmania* promastigotes and amastigotes (4×10^7^ cell equivalents) probed with anti-MCM4 antibodies and anti-tubulin antibodies (Zymed Laboratories; 1∶2000 dilution; loading control). **E**. Western blot analysis (10% SDS-PAGE) of whole cell extracts from *Leishmania* procyclics and metacyclics (4×10^7^ cell equivalents) probed with anti-MCM4 antibodies and anti-tubulin antibodies (loading control).

The MCM4 gene was amplified from *Leishmania donovani* genomic DNA using primers that were designed based on the *Leishmania major* genome sequence [16] as sequence information from *Leishmania donovani* was not available at the time. The 2.6 kb amplicon was cloned and sequenced (GenBank Accession number **GQ249892**). A comparative analysis of the MCM4 amino acid sequence with those of other eukaryotes (Figure S1) revealed that LdMCM4 shares 29–33% identity and 42–45% homology with MCM4 from other species. A stretch of approximately 80 amino acids towards the C-terminal end of LdMCM4 is absent in the other eukaryotes. Conversely, at the N-terminal region a sequence of about 85 amino acids in length that is present in other eukaryotes, is absent in LdMCM4. Like all the members of the MCM family, LdMCM4 carries the ∼200 amino acid MCM box (Figure 1A), although within this domain there is a short region of sequence insertion (13 amino acids in length) in comparison with other eukaryotes. Together, there are seven regions of sequence insertions/deletions in LdMCM4, when compared to the other eukaryotes. The MCMs are helicases belonging to the AAA+ family (ATPases Associated with various cellular Activities) and have been classified as members of the Superfamily 6 of helicases [29]. LdMCM4 carries all the signature motifs of this superfamily of helicases – the Walker A, Walker B, arginine finger, sensor 1, sensor 2 and zinc finger motifs (Figure 1A). The Walker A, Walker B and arginine finger motifs are universal structural elements involved in the binding and hydrolysis of NTPs. The MCM2/3/5 family domain of this protein spans the entire AAA module.

To evaluate the expression of MCM4 in *Leishmania*, antibodies were raised against the purified recombinant protein. The ∼97 kDa protein was expressed in *E.coli* and purified by Strep Tactin chromatography (Figure 1B). Polyclonal antibodies were raised in mice, and the specificity and sensitivity of the antibodies determined by Western blotting using recombinant LdMCM4. Anti-MCM4 antiserum detected as little as 1 ng LdMCM4 (Figure 1C) at a dilution of 1∶5000. MCM4 expression in exponentially growing *Leishmania* promastigotes was examined in whole cell extracts by Western blotting. Two bands were detected on Western blot analysis; both bands were near the expected size (Figure 1D). Neither pre-immune serum nor secondary antibody alone interacted with any proteins in whole cell extract (data not shown). The relative intensities of the two bands were somewhat variable, with some preparations having both bands of equal intensity, and other preparations having predominantly the upper band. These data suggested that MCM4 was well expressed in *Leishmania* promastigotes, and perhaps existed in two different states related to post-translational modification. Possibly, the post-translational modification(s) were partially lost during isolation, thus accounting for variabilities in the relative distribution of the two species from preparation to preparation. Analyses of MCM4 expression in logarithmically growing and stationary phase promastigotes, as well as in amastigotes, revealed that the protein was robustly expressed in all three stages, though comparatively less so in amastigotes (Figure 1D). MCM4 expression was analyzed in procyclics and metacyclics as well, and was found to be similarly expressed in both stages of promastigotes (Figure 1E).

### Analysis of possible phosphorylation of MCM4

**Figure 2:**
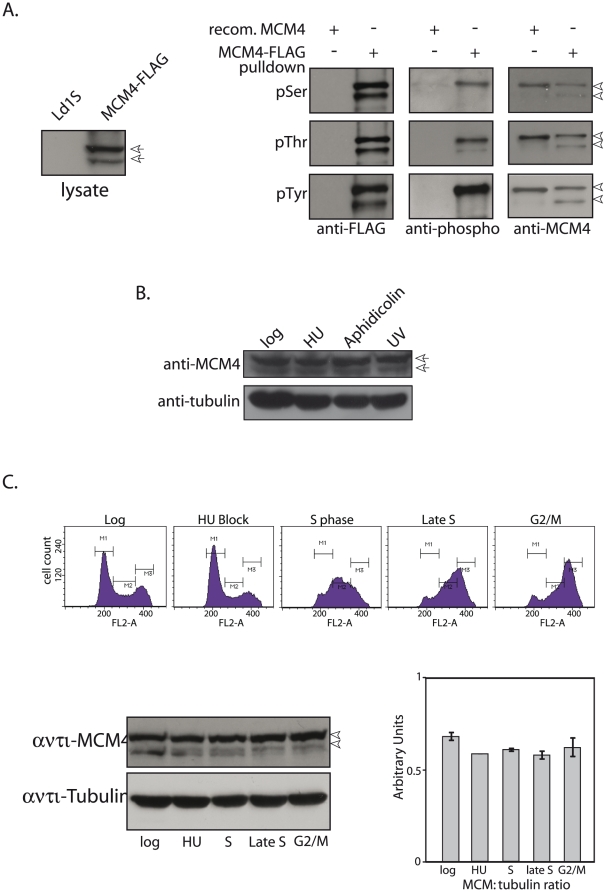
MCM4 is phosphorylated at serine, threonine and tyrosine residues, and its expression is not regulated by replication inhibitors or damage inducing agents. **A**. Analysis of phosphorylation status of immunoprecipitated MCM4-FLAG by Western blot. Left panel – Whole cell lysates made from 4×10^7^cell equivalents probed with anti-FLAG antibody (1∶5000). Right panel – Upper row: analysis for phosphoserine residues; middle row: analysis for phosphothreonine residues; lower row: analysis for phosphotyrosine residues. Antibodies and dilutions are detailed in Methods.. Arrowheads indicate MCM4-FLAG. **B**. Effect of aphidicolin and UV irradiation on MCM4 expression. Western blot analysis of extracts from 4×10^7^ cell equivalents (10% SDS-PAGE), analyzed using anti-MCM4 antibody (1∶1000 dilution) or anti-tubulin antibody (Zymed Laboratories; 1∶5000 dilution; loading control). Arrowheads – MCM4. **C**. MCM4 expression in synchronized cells. Upper panel – Flow cytometry analyses of cells harvested at different times. Lower panel - Western blot analysis (10% PAGE) of whole cell extracts (6×10^7^ cell equivalents) using anti-MCM4 antibody (1∶1000 dilution). Bar chart represents expression of MCM4 relative to tubulin. Arrows/arrowheads indicate MCM4.

To investigate the possibility of one or both forms of MCM4 being phosphorylated, we expressed MCM4 in fusion with a FLAG tag which allowed us to use FLAG M2 agarose beads to immunoprecipitate the expressed protein from transfected promastigotes. For this, promastigotes were transfected with plasmid pXG/MCM4-FLAG, and whole cell lysates of transfectant cells were first analyzed by Western blotting using anti-FLAG antibody to ascertain expression of MCM4-FLAG (Figure 2A; left panel). Thereafter, MCM4-FLAG was immunoprecipitated from the lysates and analyzed for phosphorylation status of MCM4 as described in Methods. Our analysis using phospho-antibodies revealed that MCM4 was phosphorylated on serine, threonine and tyrosine residues (Figure 2A; right panel). Interestingly, while the phosphothreonine antibodies reacted with both band species of MCM4, the phosphoserine and phosphotyrosine antibodies only interacted with the upper band of the two forms of MCM4, indicating the upper band species to be the predominantly phosphorylated form.

The expression of endogenous MCM4 in promastigotes in the presence of DNA replication inhibitors hydroxyurea and aphidicolin, as well as MCM4 expression in response to UV irradiation, was analyzed by Western blot analysis of whole cell lysates made from promastigotes that had been treated as described in Methods. We found that the expression of MCM4 did not change in response to the treatments (Figure 2B). The relative distribution pattern of both MCM4 band species also remained unaffected by the treatments. This is contrary to what is seen in mammalian cells, where MCM4 is hyperphosphorylated in the presence of DNA synthesis inhibitors or in response to UV irradiation [30], perhaps reflecting the fact that cell cycle checkpoints operating in trypanosomatids are different from other eukaryotes.

### MCM4 is expressed at more or less equivalent levels throughout the cell cycle

As the MCM2-7 complex is an essential component of the pre-RCs, we investigated the possibility of MCM4 expression being regulated through the different phases of the cell cycle, by synchronizing cells. *Leishmania* promastigotes were blocked at G1/early S by hydroxyurea treatment and then released into S phase in drug free medium. Cells harvested at different time intervals after release were analyzed for cell cycle progression by flow cytometry. Whole cell extracts were made (6×10^7^ promastigotes per time-point), and analyzed for endogenous MCM4 expression. The cells progressed through S phase and reached G2/M fairly synchronously, and MCM4 was robustly expressed throughout the cell cycle at more or less equivalent levels (Figure 2C). The phosphorylated form was expressed throughout the cell cycle (Figure 2C).

### MCM4 is constitutively located in the nucleus

**Figure 3:**
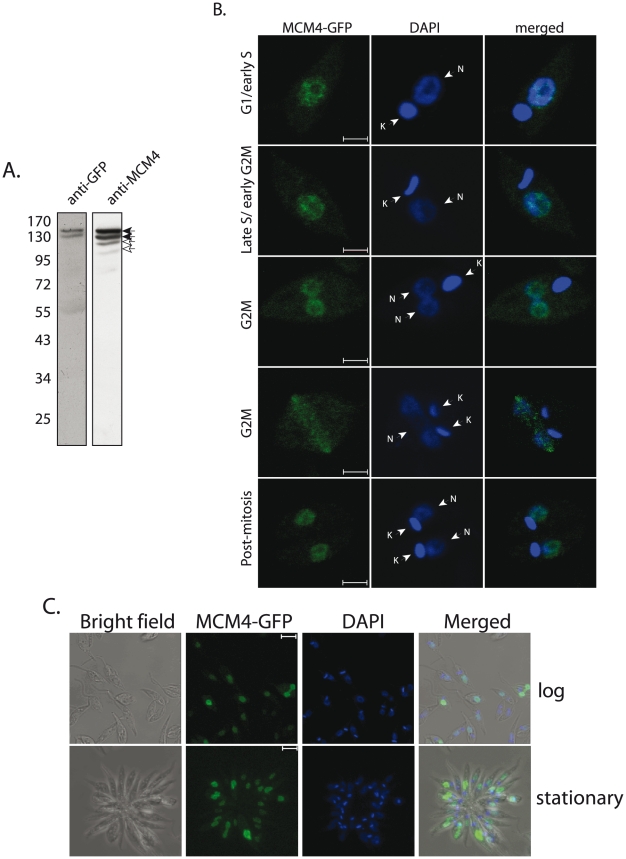
Analysis of MCM4-GFP expression in *Leishmania* promastigotes. **A**. Western blot analysis of extracts from 5×10^7^ cell equivalents (8% SDS-PAGE), analyzed using anti-GFP (1∶1000 dilution; Invitrogen) or anti-MCM4 antibody (1∶1000 dilution). Filled arrowheads – MCM4-GFP; open arrowheads – endogenous MCM4. **B**. Immunofluorescence analysis. G1/early S phase cells – one nucleus, one short kinetoplast. Late S phase/early G2/M cells – one nucleus, one elongated kinetoplast. Late G2/M phase cells - two nuclei, one kinetoplast or one nucleus, two kinetoplasts. Post-mitosis – two nuclei, two kinetoplasts. Cells were analyzed by collecting Z stack images using a confocal microscope. Magnification bar represents 2 µm. N-nucleus; K- kinetoplast. **C**. Immunofluorescence analysis of MCM4-GFP expression in logarithmically growing and stationary phase promastigotes. Magnification bar represents 5 µm.

In *S. cerevisiae* the MCMs enter the nucleus in late mitosis and remain nuclear through G1. Although detected in the nucleus to some extent in S phase, they are predominantly cytoplasmic in S phase, G2 and early mitosis [31], [32]. This nuclear exclusion is believed to prevent re-replication occurring in the same cycle. To examine the possibility of this mode of regulation operating in *Leishmania* we analyzed MCM4 expression through different stages of the cell cycle by immunofluorescence, using the kinetoplast morphology as a marker for cell cycle progression [27], [33]. We were unable to use the anti-MCM4 antibodies that we raised as they did not work in immunoprecipitations or immunofluorescence experiments. Therefore, *Leishmania donovani* promastigotes were transfected with pXG/MCM4-GFP (see Supporting Information S1) and examined for direct fluorescence of MCM4-GFP. Western blotting of whole cell lysates made from transfected cells (Figure 3A) revealed that the full length MCM4-GFP was expressed. To resolve the GFP tagged protein from the native endogenous protein, lysates were analyzed on 8% PAGE. Anti-GFP antibodies interacted mainly with MCM4-GFP protein (Figure 3A; first panel). Anti-MCM4 antibodies interacted with both MCM4-GFP and endogenous MCM4 (Figure 3A; second panel). As apparent from Figure 3A (second panel), MCM4-GFP was present at considerably higher levels than the endogenous protein.

We staged the parasites at different stages of the cell cycle using the kinetoplast shape and morphology as a marker, based on both, work published with *Trypanosoma* [33], as well as studies in our laboratory where we have microscopically compared kinetoplast morphology at different cell cycle stages using synchronized cells [27]. We found that MCM4 remains nuclear throughout the cell cycle and is not detected in the cytoplasm at any stage (Figure 3B; details of kinetoplast morphology related to cell cycle stage are in the figure legend). The expression of MCM4 was assessed in actively dividing as well as stationary phase *Leishmania donovani* promastigotes by immunofluorescence analysis of MCM4-GFP transfected cells. Rosette formation is a hallmark of stationary phase, and as is seen in Figure 3C, MCM4-GFP was well expressed in both, logarithmically growing as well as stationary phase promastigotes, and remained nuclear in both stages.

**Figure 4:**
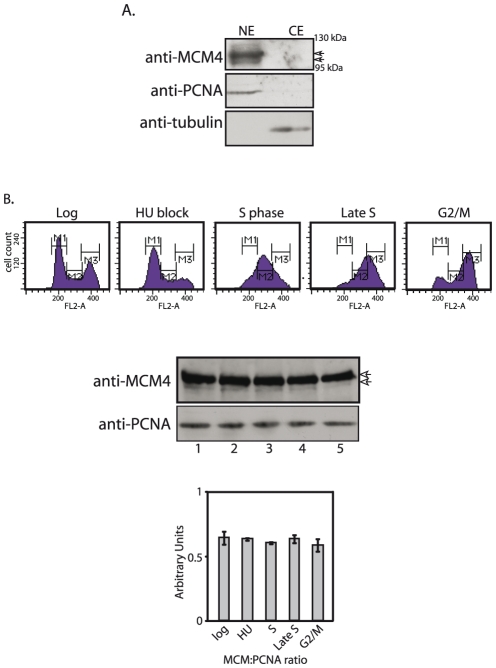
Analysis of endogenous MCM4 expression in *Leishmania* promastigotes. **A**. Western blot analysis of extracts from proliferating promastigotes. Extracts (10% SDS-PAGE) probed with anti-MCM4 antibody (1∶1000 dilution), anti-PCNA antibody (1∶5000 dilution; loading control for nuclear extracts), anti-tubulin antibody (1∶2000 dilution; loading control for cytosolic extracts). CE- cytosolic extract; NE- nuclear extract. **B**. MCM4 expression at different stages of the cell cycle. Upper panel – Flow cytometry profiles of cells harvested at different timepoints. Lower panel - Western blot analysis of nuclear extracts (4×10^7^ cell equivalents) using anti-MCM4 antibody (1∶1000 dilution) and anti-PCNA antibody (1∶5000 dilution; loading control). 1- log; 2- HU block; 3- S phase; 4- late S phase; 5- G2/M phase. Bar chart represents expression of MCM4 relative to PCNA. Arrowheads indicate MCM4.

The localization of MCM4 was also checked by Western blot analysis of cytosolic and nuclear extracts of logarithmically growing promastigotes, using anti-MCM4 antibodies. The protein was primarily nuclear in nature (Figure 4A). To reinforce the results from our immunofluorescence studies we examined synchronized cells. *Leishmania* promastigotes were synchronized using hydroxyurea, and cells harvested for flow cytometry analysis as well as preparation of nuclear extracts, at different time intervals. Extracts were prepared from equal numbers of cells (4×10^7^), and equivalent amounts of extracts were analyzed. The protein was nuclear at all times corresponding to the different stages of the cell cycle (Figure 4B), and the phosphorylated species remained in the nucleus throughout. These data indicate that subcellular localization of MCM4 is not a mode of replication regulation in *Leishmania*. In this, *Leishmania* resembles higher eukaryotes where the bulk of the MCM2-7 proteins have been reported to be nuclear throughout the cell cycle (reviewed in [34]).

### Overexpression of MCM4 in Leishmania promastigotes accelerates S phase progression

**Figure 5:**
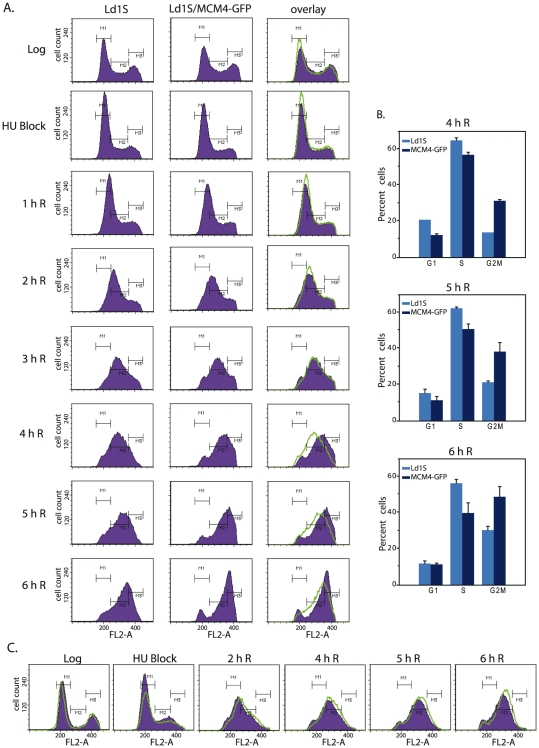
Analysis of cell cycle progression in *Leishmania* promastigotes. **A**. Flow cytometry analysis of Ld1S and Ld1S/MCM4-GFP. Data from 25000 events was collected for each time-point. In overlay histogram panels, solidly filled histograms - Ld1S/MCM4-GFP; green line - Ld1S histogram. **B**. The percent of cells at 4 h, 5 h and 6 h after release, that display the different stages of cell cycle (no significant differences were seen at earlier time-points), determined using the CellQuest Pro software. **C**. Flow cytometry analysis of Ld1S and Ld1S/GFP – overlay histograms. Solidly filled histograms - Ld1S/GFP; green line - histogram of Ld1S. G1, S and G2/M phases are indicated on the histograms by the gates M1, M2 and M3 respectively.

The impact of MCM4 overexpression on the *Leishmania* cell cycle was examined by synchronizing promastigotes and monitoring their navigation through S phase. *Leishmania donovani* promastigotes and *Leishmania donovani* promastigotes overexpressing MCM4-GFP were simultaneously blocked at G1/early S by hydroxyurea treatment and then released into S phase. Cells were harvested every hour for 6 hours and analyzed for cell cycle progression by flow cytometry. The experiment was done thrice, and histograms from one experiment are shown in Figure 5A. No significant differences in cell cycle progression were apparent in the early time-points after release; thus, MCM4 overexpression did not lead to early entry into S phase. However, cells overexpressing MCM4-GFP moved through S phase significantly faster than non-transfected Ld1S, as detected 4 h, 5 h and 6 h after release (Figure 5A; overlays of Ld1S and Ld1S/MCM4-GFP). While some variability in cell cycle progression does occur from experiment to experiment, in all three experiments performed we consistently observed that MCM4-GFP expressing cells moved ∼60 min faster than non-transfected cells.

**Figure 6:**
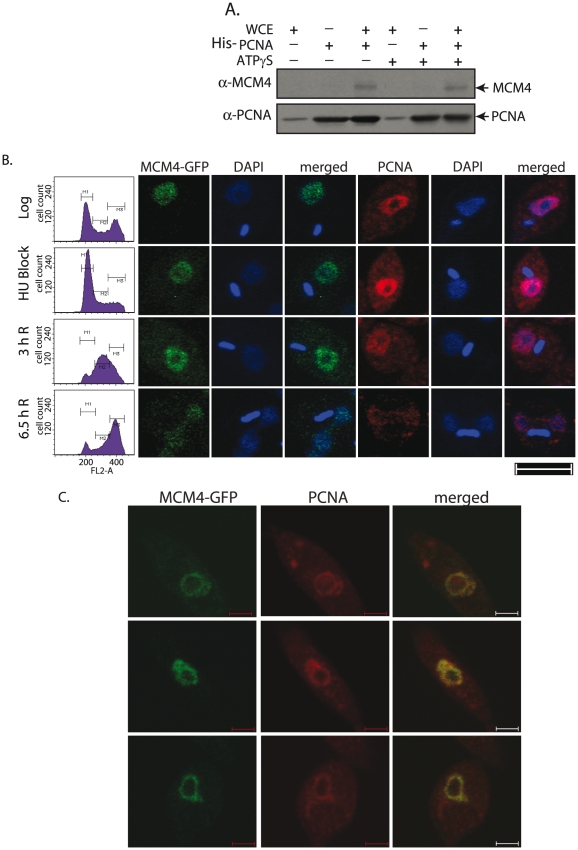
*Leishmania* MCM4 interacts with PCNA and colocalizes with it in S phase. **A**. MCM4 - PCNA interaction in pull-down experiments. Lanes 1–3 – without ATPγS; lanes 4–6 – with ATPγS. Lanes 1 & 4 – dummy metal affinity beads exposed to *Leishmania* whole cell extracts and elution carried out with imidazole. Lanes 2 & 5 – recombinant immobilized His-PCNA eluted with imidazole. Lanes 3 & 6 – recombinant immobilized His-PCNA exposed to *Leishmania* whole cell extracts, and eluted with imidazole. WCE- whole cell extracts. The PCNA band detected in lanes 1 and 4 is probably due to background PCNA from the *Leishmania* whole cell extracts poured on the beads. **B**. Immunofluorescence analysis of MCM4-GFP and PCNA expression in synchronized cells. MCM4-GFP expression was analyzed by direct fluorescence. PCNA expression was analyzed by indirect fluorescence using anti-PCNA antibodies. Magnification bar represents 5 µm. **C**. MCM4 colocalizes with PCNA in S phase cells. MCM4-GFP transfectant *Leishmania* promastigotes synchronized with hydroxyurea and harvested three hours after release were labeled for PCNA immunofluorescence as described. Cells were analyzed by collecting Z stack images using a confocal microscope. Magnification bar represents 2 µm.

Analysis of the cell cycle profiles of Ld1S and Ld1S/MCM4-GFP by CellQuest Pro software (Figure 5B; 4 h, 5 h and 6 h timepoints) revealed that cells reached G2/M phase earlier in the case of Ld1S/MCM4-GFP as compared to Ld1S, suggesting that *Leishmania* MCM4 regulates S phase of cell cycle. To investigate the possibility of the presence of G418 drug in the medium impacting cell cycle progression in case of MCM4-GFP transfected cells, and to rule out alterations in cell cycle progression due to GFP tag on MCM4, we transfected promastigotes with pXG-/GFP+ (vector carrying G418 selection marker and expressing GFP protein), and analysed them for cell cycle progression in comparison with Ld1S. As seen in Figure 5C, in fact, the cells expressing GFP under G418 selection pressure moved through S phase a little slower, signifying that in the case of MCM4-GFP transfected promastigotes, the presence of drug G418 in the medium or the presence of GFP tag is not responsible for acceleration through S phase.

### MCM4 interacts with PCNA

While DNA repair proteins, as well as many components of the replication machinery, interact with PCNA [35], none of the ORCs1-6 or MCM2-7 have been demonstrated to bind to PCNA in higher eukaryotes. *Plasmodium* ORC1 interacts with PCNA, and this interaction appears to be essential for cell viability [6], [7]. This was demonstrated in cross-species complementation experiments using a *S. cerevisiae* ORC1 swapper strain, where a yeast/*Plasmodium* chimeric ORC1 construct (wild type) could complement the mutant yeast strain, while yeast/*Plasmodium* chimeric ORC1 construct harboring PIP domain mutations of *Plasmodium* ORC1, failed to carry out complementation [6]. Several proteins that interact with PCNA are typified by the presence of the PIP box motif (**Q**xx**L/M**/**I**xx**F**/**YF/Y**) [35]. PCNA, which recruits several vital components of the replication machinery, interacts with these proteins through the same interaction domain, indicating that the interactions occur at different points in time during DNA replication. Analysis of the LdMCM4 sequence uncovered the presence of a putative PIP box at position 231–237 (**Q**HN**L**SL**Y**; Figure 1A; Figure S1). Upon analyzing the sequences of the other MCM2-7 proteins in the *Leishmania major* whole genome database we found that other than MCM4, MCM2 and MCM7 also harbor putative PIP boxes. Analysis of MCM4 in other eukaryotes revealed the presence of PIP box in MCM4 of *S. pombe*, *D. melanogaster*, *X. laevis* and *H. sapiens* (Figure S1). No PIP box was apparent in *S. cerevisiae* MCM4. We investigated the possibility of a direct interaction between MCM4 and PCNA in GST pulldown experiments, with immobilized GST-PCNA and recombinant His-tagged MCM4, in a Tris-based buffer containing either ATP, or ATPγS, or no ATP. No direct interaction was detectable (data not shown). This was not surprising as, *in vivo,* MCM4 does not act by itself, but rather, as part of the hexameric MCM2-7 complex. Also, the possible requirement of specific post-translational modification(s) (PTMs) in MCM4 for an interaction with PCNA, could not be ruled out. We therefore explored the prospect of PCNA interacting with MCM4 from *Leishmania* lysates, where it would be likely to complex with the other members of MCM2-7, and would carry PTMs as well.

Immobilized His-tagged PCNA was exposed to *Leishmania* whole cell extracts, to allow for binding of native MCM2-7 complex in a Tris-based buffer. The binding was carried out both, in the absence of ATP, and in the presence of ATPγS. As seen in Figure 6A, MCM4 was pulled down by PCNA. The interaction detected, though weak, was seen in the absence of ATP, as well as in the presence of ATPγS. This data suggests the possibility that MCM4 interacts with PCNA when it is part of the entire MCM2-7 complex, although we cannot further explore this possibility because of the lack of suitable antibodies. The interaction does not appear to be ATP-dependent, and as is apparent from the data in Figure 6A, the detected interaction is not stoichiometric, as PCNA is far in excess of the detected MCM4. A possible reason for this could be that in the absence of post-translational modifications (PTMs), the recombinant PCNA does not adopt an appropriate conformation for stable MCM4-PCNA interaction.

### MCM4 colocalizes with PCNA in S phase nuclei

Having established the existence of foci as the sites of active DNA replication in *Leishmania* cells, and demonstrating the use of PCNA as a marker for these foci [20], we investigated whether MCM4 localized to these sites in immunocolocalization studies of PCNA and MCM4-GFP. The possible colocalization of MCM4-GFP and PCNA in logarithmically growing MCM4-GFP transfectant promastigotes was examined. Most cells in such a population are in G1 phase, and results obtained clearly indicated that MCM4-GFP does not colocalize with PCNA in G1 cells (data not shown). We then synchronized MCM4-GFP transfectant *Leishmania* promastigotes by hydroxyurea treatment followed by release into drug-free medium, and sampled cells at different time intervals. Both, MCM4-GFP and PCNA localized to the nucleus at all timepoints sampled (Figure 6B), re-inforcing the fact that both these proteins are nuclear throughout the cell cycle.

We examined cells in S phase for colocalization of MCM4 and PCNA (three hours after release) by collecting Z stack images of the cells. Cells released into S phase after an eight hour hydroxyurea treatment did not have as distinct foci as do S phase cells in an asynchronous population. Hydroxyurea treatment results in a diminution of dNTP pools and this may possibly negatively impact the formation of replication foci, a feature that has been reported earlier [7]. We found that MCM4-GFP colocalized with PCNA (Figure 6C). However, to ascertain that this is not due to overexpression of MCM4-GFP, the immunolocalization pattern of the endogenous MCM4 with respect to PCNA will have to be investigated. We have been unable to do so as the anti-MCM4 antibodies do not interact with MCM4 in immunofluorescence experiments.

### The PIP box domain is important for cell viability and mediates the interaction of MCM4 with PCNA

**Figure 7:**
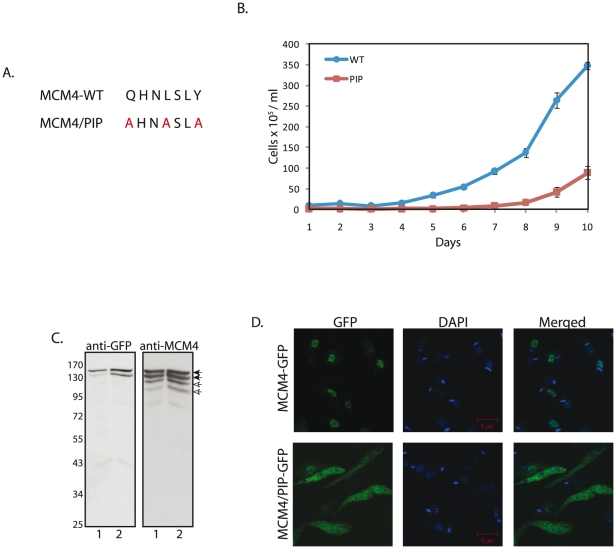
Analysis of the importance of the PIP box domain. **A**. Creation of MCM4 PIP-box mutant. The mutated residues are indicated in red. **B**. Growth analysis of strains expressing MCM4-GFP and MCM4/PIP-GFP. Closed circles – MCM4-GFP; closed squares – MCM4/PIP-GFP. Cells were counted every 24 hours. Error bars indicate standard deviation. **C**. Western blot analysis of whole cell extracts made from MCM4/PIP-GFP transfectant cultures. Extracts were analyzed on 8% SDS-PAGE by probing with anti-GFP (1∶1000; Invitrogen) or anti-MCM4 (1∶1000) antibody. 1- MCM4-GFP (wild type); 2- MCM4/PIP-GFP mutant. Filled arrowheads - MCM4-GFP, open arrowheads - endogenous MCM4. **D**. Immunofluorescence analysis of promastigotes overexpressing MCM4-GFP and MCM4/PIP-GFP. Cells were examined for direct fluorescence of the protein, 12-14 days after drug-induced selection pressure. Magnification bar represents 5 µm.

Most proteins that interact with PCNA do so via the PIP box motif (**Q**xx**L/M**/**I**xx**F**/**YF/Y**) [35]. The importance of the PIP box motif of MCM4 in mediating the MCM4-PCNA interaction, as well any possible *in vivo* role of this motif, was investigated by creating an MCM4 mutant and overexpressing it in *Leishmania* promastigotes. The PIP box in LdMCM4 (**Q**HN**L**SL**Y**) was mutated to **A**HN**A**SL**A** to create MCM4/PIP (Figure 7A). The mutant gene was cloned into the pXG vector, and the resultant plasmid pXG/MCM4/PIP-GFP was transfected into *Leishmania* promastigotes, along with control pXG/MCM4-GFP transfection. We found the pXG/MCM4/PIP-GFP transfected cultures to behave quite differently from (wild type) pXG/MCM4-GFP-transfected cultures.

The number of survivors in pXG/MCM4/PIP-GFP transfections after 6-8 days of drug induced selection pressure was 4–5 fold lower than in pXG/MCM4-GFP transfections (Figure 7B). Four separate transfection experiments of the wild type and PIP mutant constructs yielded similar results. Microscopic analysis revealed that unlike pXG/MCM4-GFP transfections, where after 6–8 days of drug-induced selection pressure more than 80–85% surviving promastigotes are MCM4-GFP positive, 90% of the few survivors detected in pXG/MCM4/PIP-GFP transfections after 6–8 days of drug induced selection pressure were MCM4-GFP negative. The decreased number of viable cells seen in case of MCM4/PIP-GFP transfectant cultures in comparison with MCM4-GFP (wild type) transfectant cultures underlines the importance of the MCM4 PIP domain in modulating cell survival and viability. After 12–14 days of selection pressure, whole cell lysates made from transfectant cultures were analyzed by Western blotting using anti-GFP as well as anti-MCM4 antibodies. We found that the full length mutant protein was being expressed in surviving cells, similar to the wild type MCM4-GFP (Figure 7C), although we have no way to find out if MCM4/PIP-GFP can form part of the MCM2-7 holocomplex due to non-availability of suitable antibodies.

The surviving transfectants were examined microscopically along with MCM4-GFP transfectants also sampled 12–14 days after selection pressure. While almost all MCM4-GFP transfectants displayed nuclear expression of MCM4-GFP, about 1 in 3000 cells displayed robust MCM4-GFP expression throughout the cell. The reason for this is unclear, but it is possible that these cells comprise a small population where MCM4-GFP for some reason does not associate as part of the MCM2-7 holocomplex and therefore localizes differently than is usual. In MCM4/PIP-GFP transfectants that survived after 12–14 days, in sharp contrast to MCM4-GFP transfectants, almost 80% cells exhibited expression of the protein throughout the cell (Figure 7D). These cells could belong to the 1 in 3000 category seen in MCM4-GFP transfectants. 20% of the cells sampled 12–14 days after drug induced selection pressure still displayed nuclear expression of the mutant protein. As two of the other MCMs that are part of the MCM2-7 holocomplex also have PIP boxes (MCM2 and MCM7) there may be some amount of functional redundancy. Hence, even though mutant MCM4 can no longer interact with PCNA, the holocomplex may be able to, via MCM2 and MCM7. To determine if the dual distribution patterns of MCM4/PIP-GFP (cytosolic in 80% cells; nuclear in 20% cells) could be segregated, we generated MCM4-GFP and MCM4/PIP-GFP clonal lines. All MCM4-GFP clonal lines displayed constitutive nuclear expression of MCM4-GFP as is seen in Figure 2B & 6C (data of clonals available on request). All MCM4/PIP-GFP clonal lines (14 clonals were analyzed) displayed the dual phenotype seen in Figure 7D (data of clonals available on request). Thus, there was phenotypic heterogeneity even within clonals. Repeated transfections with pXG/MCM4/PIP-GFP yielded similar results. These data suggested that possibly, most cells where MCM4/PIP-GFP was overexpressed did not survive, which may be the reason for the overall decrease in viability of the MCM4/PIP-GFP transfected cultures.

**Figure 8:**
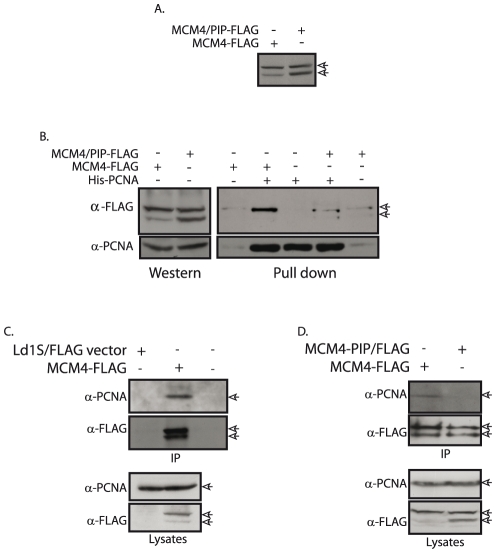
Mutations in the MCM4 PIP box domain abolish interaction of MCM4 with PCNA. **A**. Western blot analysis of MCM4-FLAG and MCM4/PIP-FLAG. Whole cell lysates from transfectant cultures (Day 14) were analyzed on 10% SDS-PAGE by probing with anti-FLAG antibody (1∶5000 dilution). Lane 1 - MCM4-FLAG (wild type); lane 2 - MCM4/PIP-FLAG mutant. Arrows indicate MCM4-FLAG proteins. **B**. Analysis of the interaction of PCNA with MCM4-FLAG and MCM4/PIP-FLAG, in pull-down experiments using recombinant His-PCNA. Left panel – Western blot analysis of input lysates with anti-FLAG antibody (1∶5000 dilution) and anti-PCNA antibody (1∶5000 dilution) to assess expression of MCM4-FLAG (∼97.2 kDa) and endogenous PCNA (∼32.4 kDa). Right panel - Western blot analysis of pulldown reactions. Lanes 1 and 5 - dummy metal affinity beads exposed to *Leishmania* whole cell extracts (MCM4-FLAG and MCM4/PIP-FLAG respectively) and elution carried out with imidazole; lanes 2 and 4 - recombinant immobilized His-PCNA exposed to *Leishmania* whole cell extracts (MCM4-FLAG and MCM4/PIP-FLAG respectively), and eluted with imidazole. Lane 3- immobilized His-PCNA only. Upper panels – immunoblot with anti-FLAG antibody to detect MCM4-FLAG proteins, lower panels- immunoblot with anti-PCNA antibody to detect His-PCNA. (Input lysates shown in left panel and pulldowns shown in right panel, were resolved on the same gel and probed with antibodies to allow alignments of bands). Arrows indicate MCM4-FLAG proteins. **C**. Analysis of MCM4-FLAG immunoprecipitates for interacting PCNA. Upper panels – analysis of immunoprecipitates with anti-FLAG antibody (1∶5000 dilution) and anti-PCNA antibody (1∶5000 dilution). Lane 1- Ld1S transfected with vector only; lane 2 – MCM4-FLAG transfectants; lane 3 – beads only control. Lower panels – Western blot analysis of input lysates for MCM4-FLAG (∼97.2 kDa) and endogenous PCNA (∼ 32.4 kDa) expression. Arrows indicate endogenous PCNA and MCM4-FLAG proteins. **D**. Analysis of the interactions of PCNA with MCM4-FLAG and MCM4/PIP-FLAG, in immunoprecipitations of MCM4-FLAG proteins using FLAG M2 agarose beads. Upper panels – immunoprecipitations of MCM4-FLAG proteins analyzed with anti-PCNA (1∶5000 dilution) and anti-FLAG antibody (1∶5000 dilution). Arrows indicate endogenous PCNA and MCM4-FLAG proteins. Lower panels – Western blot analysis of input lysates for endogenous PCNA (∼32.4 kDa) and MCM4-FLAG (∼97.2 kDa) expression. Arrows indicate endogenous PCNA and MCM4-FLAG proteins.

To assess any possible role of the PIP box motif in mediating the MCM4-PCNA interaction, the wild type MCM4 as well as MCM4/PIP mutant proteins tagged with FLAG sequence (see Supporting Information S1) were used in pulldown experiments. *Leishmania* promastigotes were transfected with the pXG/MCM4-FLAG and pXG/MCM4/PIP-FLAG plasmids. The same poor viability of transfectant cells was detected with MCM4/PIP-FLAG transfectants as was evident with MCM4/PIP-GFP transfectants. Equivalent amounts of lysates made 2 to 3 weeks after transfection from both transfectant cultures, were analyzed for MCM4-FLAG and MCM4/PIP-FLAG expression, by Western blotting using anti-FLAG antibodies. Both proteins were well expressed (Figure 8A), and therefore these lysates were used in pulldown experiments with immobilized recombinant His-PCNA. As seen in Figure 8B, MCM4-FLAG interacted with His-PCNA, behaving like the endogenous MCM4. In sharp contrast, MCM4/PIP-FLAG did not interact with His-PCNA (Figure 8B; compare MCM4-FLAG with MCM4/PIP-FLAG). The MCM4-PCNA interaction we detected was reproducible over several experiments, but not stoichiometric, in keeping with the possibility of recombinant His-PCNA conformation being non-conducive to stable MCM4-PCNA interactions, perhaps due to lack of PCNA post-translational modifications. Importantly, PCNA appears to interact only with the phosphorylated form of MCM4. This could also be the reason we were unable to detect an interaction between the two proteins, in direct pulldowns between the two recombinant proteins expressed in *E.coli*.

As the MCM4 antibodies did not work in immunoprecipitation reactions and the PCNA antibody cross-reacts with MCM4 when large amounts of extracts are used, to further investigate the MCM4-PCNA interaction we carried out immunoprecipitation of MCM4-FLAG proteins from transfected *Leishmania* cells, using FLAG M2 agarose beads, and examined if endogenous PCNA co-immunoprecipitated along with the MCM4-FLAG proteins. Initially, whole cell lysates made from *Leishmania* promastigotes harboring pXG-/GFP+/FLAG vector, as well as lysates made from *Leishmania* promastigotes harboring pXG/MCM4-FLAG plasmid, were used in immunoprecipitations with FLAG M2 agarose beads. Analysis of the immunoprecipitates by Western blot with anti-PCNA antibody revealed that endogenous PCNA co-immunoprecipitated with MCM4-FLAG (Figure 8C, lane 2). No PCNA was detected in beads only control (Figure 8C, lane 3) or empty vector transfectant control (Figure 8C, lane 1). The role of the PIP box domain in mediating the detected MCM4-PCNA interaction, was investigated by carrying out immunoprecipitations of both, MCM4-FLAG and MCM4/PIP-FLAG proteins, from lysates of transfectant cultures. As seen in Figure 8D, we found that while wild type MCM4-FLAG interacted with endogenous PCNA, the MCM4/PIP-FLAG mutant did not. These data reinforce our findings that *Leishmania* MCM4 interacts with PCNA, and indicate that the interaction is mediated by the PIP box motif of MCM4.

## Discussion

While a substantial body of literature bears testimony to the fact that DNA replication in eukaryotes is a highly conserved process, replication of the genomes of trypanosomatids has remained largely unexamined. We have endeavoured to investigate one of the replication proteins in *Leishmania donovani*, the causative agent of the deadly disease visceral Leishmaniasis. In our study we find noteworthy differences with other eukaryotes. This is not surprising as several orthologs of eukaryotic replication proteins are missing in *Leishmania*, as determined from annotation of the *Leishmania* genome sequences [16], [17]. It is possible that novel *Leishmania* proteins regulate the process in this organism.

Functional data has revealed that all six components of MCM2-7 contribute to the same activity. Immunodepletion experiments in *Xenopus* extracts indicate that depleting any single MCM protein is sufficient to negatively impact DNA replication [36]–[38]. We have cloned and characterized one of the six MCM2-7 proteins, MCM4. LdMCM4 has the conserved motifs associated with this protein (Figure 1A). The MCM2-7 belong to the family of AAA+ ATPases and LdMCM4 has the P-loop NTPase domain that characterizes members of this family (residues 437 to 589). While all the MCM2-7 belong to the AAA+ ATPase family and have ATP binding sites, individual MCM subunits do not display ATPase activity [39]. Analysis of MCM4 expression in *Leishmania* promastigote extracts revealed the presence of two bands near the expected size, and probing of MCM4-FLAG with phospho-antibodies suggests that the upper band corresponds to phosphorylated form (Figure 2A). MCM4 has been shown to be variably phosphorylated through the cell cycle in *S. cerevisiae*, *Xenopus*, and mammalian cells [40], [41], with chromatin-bound MCM4 undergoing specific phosphorylation in S phase. MCM4 phosphorylation in S phase in mammalian cells promotes the association of Cdc45 with chromatin as well [42]. We found the phosphorylated form to be dominant throughout the cell cycle (Figure 2C), and it remains nuclear throughout the cell cycle (Figure 4B). However, site-specific phosphorylation events may be modulated in cell cycle dependent manner. Site-specific phosphorylations may also modulate MCM4 activity. Much more detailed investigations need to be carried out to address these issues.

The subcellular localization of MCM4 has been demonstrated to be a mode of replication regulation in *S. cerevisiae* [31], [32], where nuclear export in S phase is believed to prevent re-replication from occurring. We found *Leishmania* to be different in that MCM4 remained in the nucleus throughout the cell cycle (Figures 3B, 4B). In this, it resembles mammalian cells, where cell cycle progression does not affect MCM4 localization. Interestingly, the overexpression of MCM4 in *Leishmania* resulted in a shortened nuclear S phase, with cells reaching G2/M faster than usual (Figure 5). This suggests the possibility that cellular MCM4 levels may be limiting, an attribute of the protein that may play a role in modulating cell cycle progression.

Investigating possible interactions between MCM4 and PCNA revealed that while purified recombinant MCM4 expressed in *E.coli* by itself does not bind to PCNA (data not shown), MCM4 in whole cell lysates interacts with PCNA (albeit somewhat weakly) (Figure 6 and Figure 8). PCNA interacts only with the phosphorylated form of MCM4 (Figure 8B), indicating this as the likely reason why we were unable to detect the interaction in direct pulldowns between the two recombinant proteins expressed in *E.coli*. It is also possible that MCM4 needs to be part of the MCM2-7 complex for the interaction to occur, although we have no experimental evidence of this. The MCM4-PCNA interaction appears to be ATP-independent, and while it is possible that the MCM4-PCNA interaction is mediated through other protein(s), we find that mutating a sequence in MCM4 that has been shown to be directly responsible for protein-PCNA interactions in other proteins (PIP box), results in the loss of the MCM4-PCNA interaction. As no other role has been assigned to this motif to date, this data suggests that the interaction is between MCM4 and PCNA (perhaps as part of the MCM2-7 holocomplex, though there is no direct evidence that the MCM4-PIP box mutant can still form a functional complex with the other members of the MCM2-7 proteins). The PIP domain is important for cell viability, as overexpression of MCM4-PIP box mutant that cannot interact with PCNA (Figure 8) results in overall decreased viability of *Leishmania* cultures (Figure7). One interesting feature we observed was that the MCM4/PIP-FLAG protein seems to have a partial phosphorylation defect. As one of the residues mutated in the PIP box is a tyrosine residue, and tyrosine phosphorylation is detected in MCM4 (Figure 2A), it is possible that this residue is a site of phosphorylation in the protein. However, much more needs to be done to ascertain this. While PCNA in other eukaryotes has been shown to interact with several proteins involved in DNA repair and replication [6], [7], [35], to date no interactions with any of the MCM2-7 have been detected in any eukaryote. In *S. cerevisiae*, MCM10 has been shown to interact with PCNA [43]. This interaction is through the MCM10 PIP box, and an MCM10 PIP box mutant displays a severely defective cell growth and proliferation phenotype. MCM10 also interacts with MCM2-7 [44]–[47], ORC [44], [46], [48]–[49] and DNA polymerase α/primase complexes [50]–[53], and promotes initiation of replication as well as elongation. The loading of PCNA onto chromatin is believed to be facilitated via its interaction with MCM10, which is loaded onto pre-RCs after MCM2-7 [43]. In *Leishmania,* the role of facilitation of chromatin loading of PCNA may be played by the MCM2-7 complex itself. Alternatively, the MCM2-7 complex and PCNA may be associated during elongation, as both proteins move along with the replication fork, MCM2-7 being the replicative helicase, and PCNA being the DNA polymerase δ processivity factor. Studies carried out by several research groups have shown that the MCM2-7 do not colocalize with PCNA in other eukaryotes [54]–[57]. However, during *Drosophila* chorion gene amplification, MCM2-7 immunocolocalized with PCNA at all stages of amplification [58] indicating that these MCMs are present at chorion amplicons through replication initiation as well as elongation of replication forks. The observation that MCM4-GFP in *Leishmania* promastigotes immunocolocalizes with PCNA in cells that are in S phase (Figure 6C), is suggestive of the possibility that the MCM2-7 move along with the elongating replication forks in *Leishmania*. A role for MCM2-7 in replication fork elongation has been demonstrated in higher eukaryotes. Chromatin immunoprecipitation experiments demonstrate that the MCMs are displaced from origins and, along with Cdc45, move ahead of the replication fork, in contrast to the ORC proteins which do not [59]. Experiments where the MCMs were destroyed after replication initiation indicated the continued necessity of MCMs in DNA replication even post-initiation [60]. The colocalization of MCM4 and PCNA in *Leishmania* is an aspect that needs to be further investigated by examining the behavior of endogenous MCM4, to rule out the possibility of our observations being the result of overexpression.

The data presented here describe the findings of an investigation of one of the key players of eukaryotic DNA replication in the protozoan *Leishmania donovani,* and lay the foundation for future studies directed at addressing the role of the MCM4-PCNA interaction in DNA replication. The results of our study underline the importance of studying DNA replication in non-conventional organisms as much as in model systems, and uncover facets of emerging diversities in modes of replication among eukaryotes.

## Supporting Information

Figure S1 **Sequence analysis of** ***Leishmania donovani*** **MCM4.** ClustalW analysis of LdMCM4 with MCM4 from other eukaryotes viewed using Jalview multiple alignment editor (Waterhouse AM, Procter JB, Martin DM, Clamp M, Barton GJ. 2009. Jalview Version 2—a multiple sequence alignment editor and analysis workbench. Bioinformatics. **25**:1189-1191). *H. sapiens, Homo sapiens; X. laevis, Xenopus laevis; D. melanogaster, Drosophila melanogaster; S. pombe, Schizosaccharomyces pombe; S. cerevisiae, Saccharomyces cerevisiae; L. donovani, Leishmania donovani.* PIP boxes are indicated with black boxes.
(EPS)

Table S1 **Sequences of oligonucleotides used in this study.**
(DOC)

Supporting Information S1 **Cloning details and preparation of** ***Leishmania*** **extracts,**
(DOC)
