# Supplementary material for: Characterization of Leishmania donovani MCM4: Expression Patterns and Interaction with PCNA
Source: PLoS One. 2011 Jul 29;6(7):e23107. doi: 10.1371/journal.pone.0023107 (PMC3146543; doi:10.1371/journal.pone.0023107)
Supplement: Table S1 — Sequences of oligonucleotides used in this study. (DOC) [file pone.0023107.s002.doc]

**Table I:** **Sequences of oligonucleotides used in this study**

| **Name** | **Sequence** |
| --- | --- |
| MCM4-F | 5’ CACCGAATTCCATATGCAGCAGCGCTCGGAGGACTAC 3’ |
| MCM4-R | 5’ TCGGGATCCTGCCATTGGCGCTGCTCCGGCCATCGC 3’ |
| MCM4-GFP-F | 5’ CACCGGATCCACCATGCAGCAGCGCTCGGAGGACTAC 3’ |
| MCM4-GFP-R | 5’ TCGATATCTCCCATTGGCGCTGCTCCGGCCAT 3’ |
| MCM4-PIP-F | 5’ AAGCTGGCGCACAACGCGTCGCTCGCCGAGGAC 3’ |
| MCM4-PIP-R | 5’ GTCCTCGGCGAGCGACGCGTTGTGCGCCAGCTT 3’ |
| FLAG-U | 5’ ATCGACTACAAGGACGACGATGACAAGTGACG 3’ |
| FLAG-L | 5’ CGTCACTTGTCATCGTCGTCCTTGTAGTCGAT 3’ |
