# Supplementary material for: Characterization of Leishmania donovani MCM4: Expression Patterns and Interaction with PCNA
Source: PLoS One. 2011 Jul 29;6(7):e23107. doi: 10.1371/journal.pone.0023107 (PMC3146543; doi:10.1371/journal.pone.0023107)
Supplement: Supporting Information S1 — Cloning details and preparation of Leishmania extracts, (DOC) [file pone.0023107.s003.doc]

**Cloning details and preparation of *Leishmania* extracts**

***Subcloning of MCM4 for expression in E.coli and Leishmania***

For overexpression in *E.coli*, *LdMCM4* was subcloned into EcoRI, BamHI sites of pASK-IBA43plus (IBA BioTAGnology). For expression in *Leishmania* promastigotes in fusion with GFP, *LdMCM4* was amplified using primers MCM4-GFP-F and MCM4-GFP-R (Table S1), and the amplicon cloned into BamHI, EcoRV sites of pXG-/GFP+ (a kind gift from the laboratory of Dr. S.M. Beverley) (1), creating plasmid pXG/MCM4-GFP. LdMCM4 was expressed in fusion with FLAG tag at the C-terminal end by first cloning the FLAG tag peptide sequence in frame into the EcoRV site of pXG-/GFP+ (creating the plasmid pXG-/GFP+/FLAG), so as to retain the EcoRV site before the tag sequence, but not after it. A stop codon introduced at the end of the tag sequence prevented expression of the downstream GFP gene. The FLAG tag sequence was generated by annealing oligonucleotides FLAG-U and FLAG-L (Table S1) and cloning into the EcoRV site of pXG-/GFP+. *MCM4* amplicon generated by MCM-GFP-F and MCM-GFP-R primers was cloned into BamHI, EcoRV sites of pXG-/GFP+/FLAG, generating plasmid pXG/MCM4-FLAG. The MCM4/PIP amplicon was cloned into pXG-/GFP+ and pXG-/GFP+/FLAG in similar fashion.

***Separation of procyclics from metacyclics***

To separate *Leishmania* procyclics and metacyclics, 4-5 x 10^8^ promastigotes were harvested from day 5 cultures, washed twice with 1X PBS, resuspended in 5 ml PBS, equal volume of PNA (Sigma Aldrich, USA) added to a final concentration of 100 µg/ml, the mix incubated for 1 h at room temperature, and the agglutinated procyclics separated from the metacyclics by centrifugation at 100*g*/3 min. The metacyclics were then harvested by centrifugation at 1000*g/*3 min. The cell pellets were directly lysed in SDS-sample loading buffer for Western blot analysis.

***Preparation of cytosolic and nuclear extracts of Leishmania***

Cytosolic and nuclear extracts were made by the method of Walker & Saravia (2) with a few modifications. ~1-10 x10^7^ promastigotes were harvested, washed twice in 1X PBS, resuspended in 400-600 µl PBS containing 0.5 mM DTT and protease inhibitors, incubated for 10 min on ice, and gently sonicated at 20% power output. Nuclei were collected by centrifuging at 16000g / 5 min (supernatant was the cytosolic fraction), and proteins extracted from the nuclei pellet using the NER reagent (Pierce Biotechnology NE-PER kit).

**References:**

1. **Ha, D. S., J. K. Schwarz, S. J. Turco, and S. M. Beverley**. 1996. Use of the green fluorescent protein as a marker in transfected Leishmania. Mol. Biochem. Parasitol. **77:**57-64.
2. **Walker, J., and N.G. Saravia.** 2004. Inhibition of *Leishmania donovani* promastigote DNA topoisomerase I and human monocyte DNA topoisomerases I and II by antimonial drugs and classical antitopoisomerase agents. J. Parasitol., **90**: 1155-1162.
